# Internal freezing and heat loss of apple (*Malus domestica* Borkh.) and sweet cherry (*Prunus avium* L.) reproductive buds are decreased with cellulose nanocrystal dispersions

**DOI:** 10.3389/fpls.2022.949537

**Published:** 2022-11-17

**Authors:** Brent Arnoldussen, Jassim Alhamid, Peipei Wang, Changki Mo, Xiao Zhang, Qin Zhang, Matthew Whiting

**Affiliations:** ^1^ Irrigated Agriculture Research and Extension Center (IAREC), Department of Horticulture, Washington State University, Prosser, WA, United States; ^2^ School of Mechanical and Material Engineering, Washington State University, Richland, WA, United States; ^3^ Voiland School of Chemical Engineering and Bioengineering, Washington State University, Richland, WA, United States; ^4^ Center for Precision and Automated Agricultural Systems (CPAAS), School of Biological Systems Engineering, Washington State University, Prosser, WA, United States

**Keywords:** cold hardiness, frost damage, calorimertry, thermography, cold damage

## Abstract

Cold damage has caused more economic losses to fruit crop growers in the U.S. than any other weather hazard, making it a perennial concern for producers. Cellulose nanocrystals (CNCs) represent a new generation of renewable bio-nanomaterials, with many unique physical and chemical properties, including their low thermal conductivity. Our team has developed a process for creating CNC dispersions that can be sprayed onto woody perennial crops, forming a thin insulating film around buds which has been shown to increase cold tolerance. Using digital scanning calorimetry (DSC) on dormant apple (*Malus domestica* Borkh.) reproductive buds, we investigated the thermodynamic properties of plant materials treated with CNC dispersion at lower temperatures. Scanning electron microscopy (SEM) was used to evaluate the thickness of the CNC films and their deposition on the sweet cherry bud surface. Apple buds treated with 3% CNC exhibited lethal freezing at temperatures 3.2°C and 5.5°C lower than the untreated control when sampled 1 and 3 days after application, respectively. Additionally, the latent heat capacity (J/g) of the 3% CNC-treated buds was 46% higher compared with untreated buds 1 day after application, and this difference increased 3 days after application to 168% higher. The emissivity of cherry buds treated with 3% CNC was reduced by an average of 16% compared with the untreated buds. SEM was able to detect the dried films on the surface of the buds 3 days after application. Film thickness measured with SEM increased with material concentration. The emissivity, HTE, and LTE results show that CNC-treated reproductive buds released thermal energy at a slower rate than the untreated buds and, consequently, exhibited internal ice nucleation events at temperatures as much as 5.5°C lower. The increased enthalpy during the LTE in the CNC-treated apple buds shows more energy released at lethal internal freezing, indicating that CNC coatings are increasing the amount of supercooled water. The effects of CNC shown during the DSC tests were increased by CNC concentration and time post-application. These results suggest that CNC dispersions dry into nanofilms on the bud surface, which affects their thermodynamic processes at low temperatures.

## 1 Introduction

Unusual weather patterns, early bud break due to warmer winter or spring temperatures, and market-driven industry expansion into higher-risk areas have led to periodic but catastrophic economic losses due to cold damage to fruit crops. During a 5-year period, which accounted for the last USDA crop insurance statistics, cold damage accounted for more than 50% ($293 million per year) of the total crop-loss insurance paid out to growers of tree fruits and grapes (Shields, 2015). Some of the most destructive cold events recorded in history include the “Easter freeze” (April 2007), “killer frost” (April 2012), and “polar vortex” (January 2014) (Wisniewski et al., 2018) and widespread freezes across Europe in 2017 which accounted for 3.4 billion euros in damage (Faust and Herbold, 2018). These events encompassed large geographic regions and led to significant crop and economic losses. Moreover, the tools available to growers have remained largely unchanged for several decades, while the risk of frost damage is increasing due to a changing climate (Unterberger et al., 2018; Miranda et al., 2019).

Industry-standard methods for reducing cold damage during a cold event rely on increasing the temperature of the orchard or plant-level microclimate with wind machines, orchard heaters, or irrigation, often in combination (Snyder and Melo-Abreu, 2005). These methods, on average, can provide up to 3°C of added tolerance during frost events. Still, these results are highly variable and depend on weather conditions such as radiative or advective freeze events, low temperature, cloud cover, and wind speed (Perry, 1998; Snyder and Melo-Abreu, 2005; Poling, 2008; Wisniewski et al., 2008). Additionally, these methods require the constant operation of the equipment that must be appropriately timed and requires significant energy usage (Cary, 1974). There has been a long history of research and grower interest in the development of a sprayable frost protectant as an alternative active frost protectant. Past research has identified several different materials that can decrease cold damage in plants. The mechanisms of frostprotection of material can generally be classified into three main modes of action. These include insulation, cryoprotection, and nucleation inhibition.

An insulative material is a common mechanism of frost protectants discussed in previous studies. These protectants decrease the rate at which the plant tissues cool by reducing heat loss to the environment. Insulative aqueous foams have been tested extensively since the 1960s (Braud and Chesness, 1970). Multiple formulations of low thermal conductivity foams have been shown to create an insulative barrier in low-growing crops such as lettuce and strawberries, offering up to 9°C protection (Choi and Giacomelli, 1999; Choi et al., 1999). However, because of application and durability constraints, these materials are best suited for low-growing crops and have not been used effectively in trees (Bartholic, 1985; Choi et al., 1999). Several other insulative materials can be used to cover the crops physically. Polypropylene films can cover low-growing row crops (Bhullar, 2012). Polyethylene bags were used to cover floral tissue or fruits of peaches (Prunus persica) and low-density polyethylene coverings for vines and tree branches (Willwerth et al., 2014).

Cryoprotectants or antifreeze compounds lower the ice nucleation temperature of the liquids inside the cells or tissues (Griffith et al., 2005). Many sprayable synthetic and antifreeze compounds have been tested in tree fruits (Rieger, 1989). These were generally high molecular weight surfactant chemicals such as ethylene glycol, polyethylene glycol, dodecyl ether of polyethylene glycol (DEPEG), and similar polymers (Himelrick et al., 1991; Wilson and Jones, 1980; Wilson and Jones, 1983). These compounds have been tested but showed inconsistent efficacy in tree fruit and grapes (Himelrick et al., 1991; Matta et al., 1987; Rieger and Krewer, 1988). While some of these materials were effective, concerns with the general toxicity of the widespread application of the compounds precluded their widespread use. Similarly, other types of molecules that can affect the osmotic and solute potential and subsequently decrease the freezing temperature of internal fluids have been explored. Foliar fertilizers and seaweed and fungal extracts have been tested as frost protectants in fruit crops with varying efficacy depending on the material, plant growth stage, and species or cultivar (Centinari et al., 2016; Centinari et al., 2018; Karimi, 2019; Karimi et al., 2022).

Finally, sprayable frost protectants can also physically impede ice nucleation and propagation. Coatings of inert particles prevent the formation or propagation of ice crystals by blocking extrinsic nucleation sites (Wisniewski et al., 2002). Extrinsic nucleation sites include ice-nucleating active (INA) bacteria, plant and microbial metabolites, and environmental impurities in freezing water. Acrylic film and kaolin clay coatings have been shown to increase the cold survivability of sensitive tissues by blocking extrinsic nucleation sites and preventing the formation of damaging ice crystals within the tissues (Glenn et al., 1998; Fuller et al., 2003). Additionally, blocking extrinsic nucleation has been shown to facilitate the tissue’s innate ability to supercool water by encouraging non-damaging ice nucleation at intrinsic nucleation sites (Wisniewski et al., 2002).

Cellulose nanocrystals are bionanomaterials composed of uniform crystalline structures of cellulose with a length of 100 to 300 nm and a width between 5 and 15 nm (Habibi, 2014). These nanocrystals can be renewably produced on an industrial scale from different plant materials such as wood pulp and agricultural byproducts (e.g., soybean, corn, sugarcane bagasse, hemp) (Siqueira et al., 2010; Costa et al., 2015; Cudjoe et al., 2017; Martelli-Tosi et al., 2018). The unique properties of nanocellulose have led to its use in many industrial sectors, including paints and coating, polymer composites, catalysis, cosmetics, biosensors, drug delivery, and medical devices (Lam et al., 2012).

Our team has recently formulated a cellulose nanocrystal (CNC) dispersion that can be applied with commercial orchard application equipment to form a thin film on the surface of dormant or developing flower buds (Zhang et al., 2021). Spray applications can offer as much as 3°C protection to both dormant grape buds and developing cherry flowers 24 h post-application (Alhamid et al., 2018). The films created by these dispersions have been shown in lab studies to have an extremely low thermal conductivity (0.061 W/m−1 K−1). We hypothesize that the dispersions create low thermal conductivity films around the buds which likely function as both an insulative barrier as well as an ice nucleation inhibitor. These combined effects increase the ability of the coated reproductive buds to withstand colder temperatures than the control.

The biophysical mechanisms of the protection provided by CNC coatings have not been demonstrated *in vivo*. A better understanding of the mechanisms involved in the increased cold tolerance of fruit reproductive tissues treated with CNC is important to understand further their utility as a frost protectant. The overall goal of this work is to better characterize the films created by CNC applications and their effects on thermal dynamic and ice nucleation properties of apple and cherry reproductive buds using calorimetry, thermography, and electron microscopy techniques.

## 2 Materials and methods

### 2.1 CNC dispersion

The raw CNC material was obtained from the USDA Forest Products Laboratory (FPL), and 2% and 3% (wt) CNC dispersions with cetrimonium bromide surfactant and a trace amount of stabilization agents were used to improve surface adhesion ability, quick wetting, and uniform coverage on plant tissues (Zhang et al., 2021). The solution was dispersed using a sonication process published previously (Alhamid et al., 2018; Zhang et al., 2021). The CNC particles used have an average length of 360 nm and width of 10 nm when measured using X-ray scattering analysis.

### 2.2 Dispersion application

Branches from the dormant apple ‘WA 38’ and sweet cherry ‘Bing’ were randomly collected from the Washington State University Roza research orchards for the calorimetry and thermal image analyses, respectively. Branches were transported to the lab in a cooler and then treated with either one of the CNC dispersions or a water control using a single nozzle electrostatic sprayer (On Target Spray Systems, Mt. Angel, OR, USA). All applications were done at ambient lab temperatures, with the CNC or water also being stored at room temperature. The sprayer flow rate was calibrated to be 180 ml/min. This type of sprayer was chosen because electrostatically charged particles improve coverage while using significantly less material (Oakford et al., 1994; Law, 2001). Plant materials were stored at 0°C after application until analysis.

### 2.3 Calorimetry

Calorimetry techniques such as digital scanning calorimetry (DSC) measure heat flow associated with thermal transitions and phase changes of material and are routinely employed to calculate the thermodynamic properties of nano-sized material and biomolecules in nanoscience (Gill et al., 2010) and the ice nucleation activity and thermal kinetics inside of woody plant tissues (George and Burke, 1977; Sugiura et al., 1995; Vetrucci and Stushnoff, 1992). The freezing of reproductive buds is well documented to produce two exothermic events during freezing due to the latent heat of fusion. The high-temperature exotherm (HTE) is a significant energy release corresponding with the non-lethal, extracellular freezing of the plant tissue, and the low-temperature exotherm (LTE) is a minor release of energy that corresponds to lethal intracellular freezing (Quamme et al., 1972; Burke et al., 1976; Proebsting and Mills, 1978).

These exothermic events are also observed in the commonly used technique of differential thermal analysis (DTA). DSC can also determine the temperature at which HTE and LTE nucleation events occur. Also, the greater accuracy of this analytical method allows for the energy released during nucleation events (latent heat capacity/enthalpy) to be more accurately quantified. This information will enable DSC to elucidate more information regarding the thermal energetics of the tissue to be understood (Burke et al., 1976; Olien and Livingston, 2006; Livingston, 2007). Whereas DTA allows for the rapid and high-throughput determination of lethal temperatures of a larger sample size of reproductive buds, making it a preferred method for determining the cold tolerance of floral buds in a field setting (Andrews et al., 1984; Mills et al., 2006), DSC is the preferred analytic approach for studying the mechanical behavior of ice formation in plant tissues.

**Figure 1:**
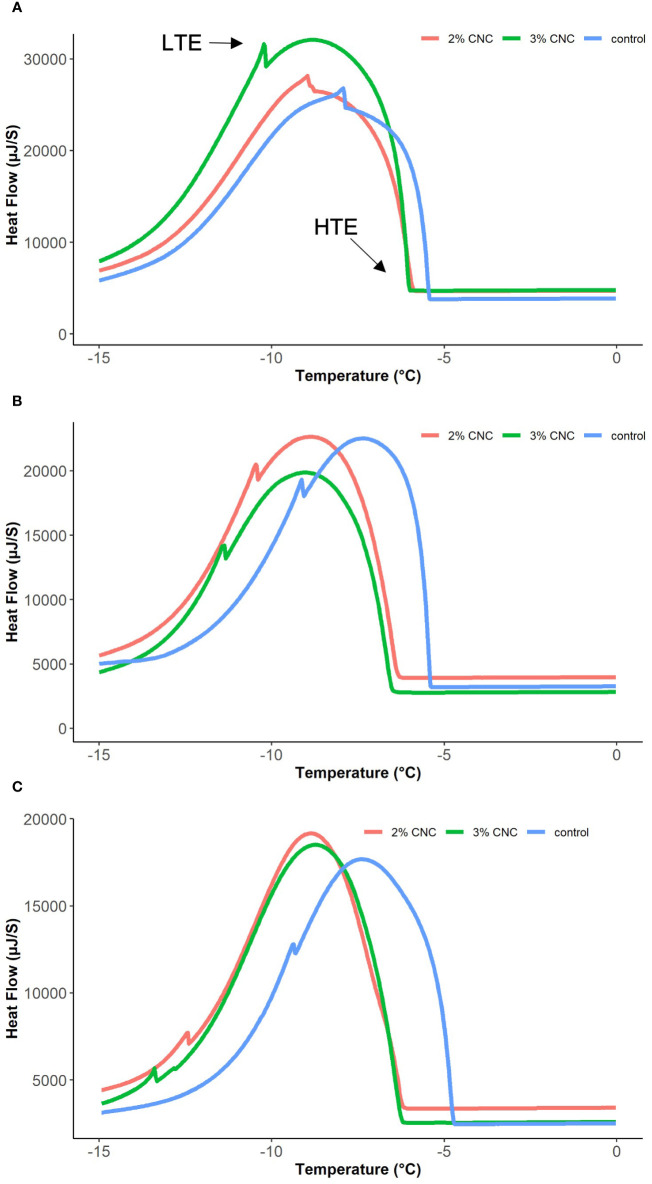
Selected heat flow curves from digital scanning calorimetry (DSC) of apple buds treated with 2% or 3% (wt) cellulose nanocrystal dispersion or water control, 1 day **(A)**, 2 days **(B)**, and 3 days **(C)** post-application.

Apple reproductive buds from each treatment (*n* = 5) were dissected from the branches, weighed, and placed into the DSC (MCDSC Model 600000, TA Instruments, USA) for scanning. Tests were conducted 1, 2, and 3 days after the CNC application. The scanning temperature was equilibrated at 5°C for 10 min before lowering to −15°C at a rate of 0.4°C/min. Heat rate (μJ/s) curves were recorded from the DSC software interface (NanoAnalyze Software v3.8.0 by TA Instruments, USA). The heat rate curves were analyzed visually to identify the temperatures corresponding to the HTE and LTE peaks and non-lethal and lethal freezing of the flower primordia within the buds (**Figure 1**). LTE peaks were integrated to determine the energy (μJ) released during nucleation, which was then corrected with the total sample weight collected immediately before testing, to derive latent heat capacity (J/g). Mean HTE, LTE, and latent heat capacity or enthalpy were compared using a generalized linear model to test the effect of treatment and time and Tukey’s honestly significant difference for mean separation in RStudio.

### 2.4 Thermography

Thermography studies using thermal infrared cameras have been influential in the study of plant freezing by allowing a visual evaluation of freezing events (Wisniewski and Fuller, 1999; Hacker and Neuner, 2008). These remote sensors can also measure tissue surface temperature (Chandel et al., 2018). Additionally, they can be used to measure the emissivity, or the ability to emit energy by radiation, of plant materials (Chen, 2015; López et al., 2012).

In general, all material objects emit electromagnetic radiation (EM), and the relation between the corresponding temperature (*T*) of an object and radiated power (*R*) is described by the Stefan–Boltzmann law.


(1) $$\text{Q}={\text{ƐT}}^{4}$$

Where *ϵ* is defined as the emissivity of the object, emissivity is reported between 0 and 1, and its value depends on the specific type of material. By definition, a blackbody has a perfect emissivity, i.e., *ϵ* = 1. Plants generally present about 0.95 (Chen, 2015). By measuring the emissivity of cherry buds treated with CNC solution, changes in the rate of radiative energy loss from the insulative effect of the coatings can be quantified and used to confirm DSC results.

**Figure 2:**
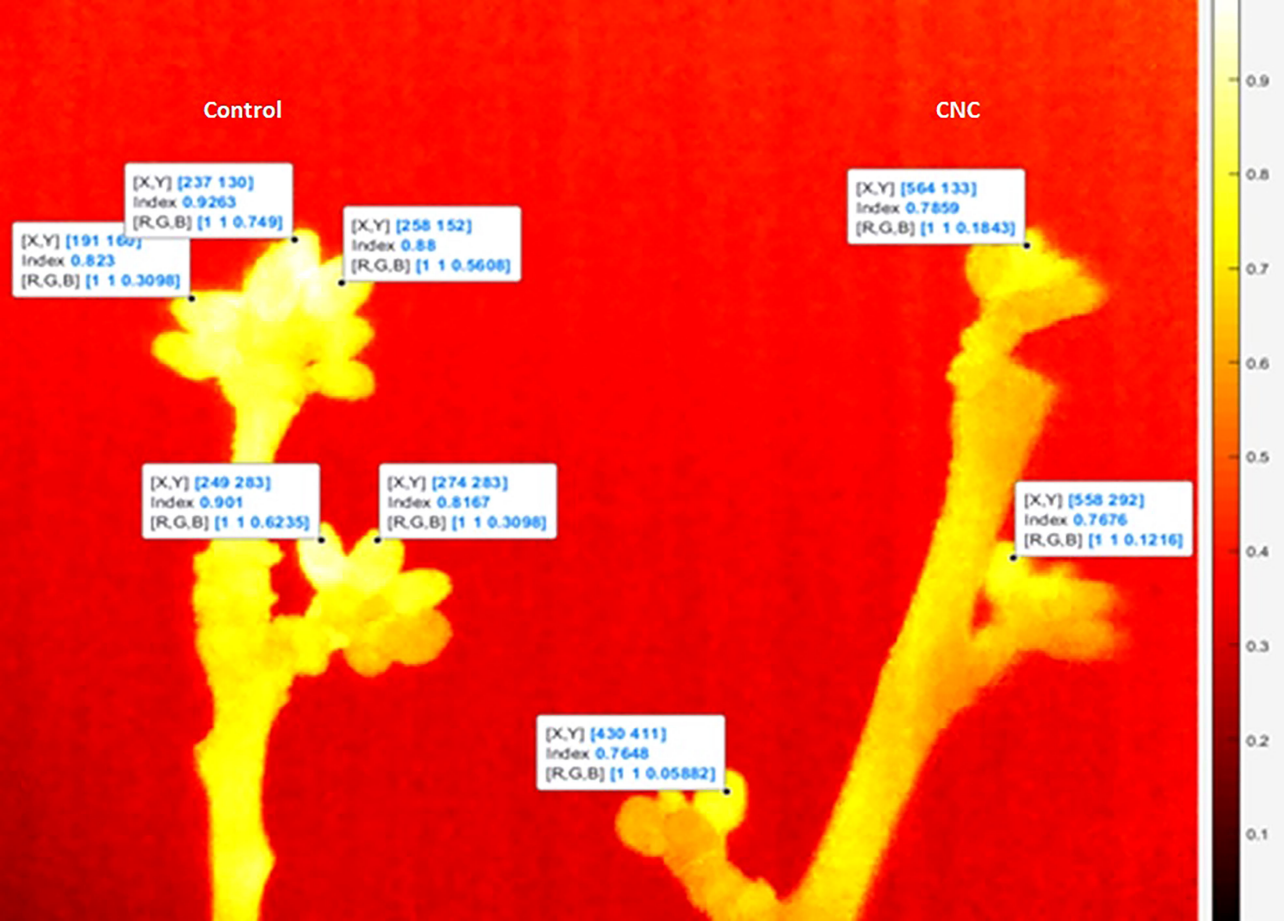
Thermography emissivity image of untreated (right) and 2% (wt) cellulose nanocrystal dispersion-treated (left) sweet cherry fruiting spurs and flower buds.

Twelve- to 17-cm segments of ‘Bing’ sweet cherry branches containing 10–14 reproductive buds (two fruiting spurs) were treated with either 3% CNC or control and then one branch of each treatment was placed into a programmable environmental chamber. The chamber temperature was equilibrated at 5°C and then decreased to −15°C at a rate of 4°C h^−1^. A FLIR TC 640 thermal camera (FLIR, Wilsonville, OR, USA) was calibrated using a blackbody with a known emissivity. The lens of the calibrated camera was then placed into the chamber to capture both branches in the field of view. Images were captured at nine different temperatures (14°C, 7°C, 0°C, −0.7°C, −3.5°C, −4°C, −6.6°C, −9.4°C, −10°C) as the chamber decreased in temperature. This process was replicated 10 times with the mean of total buds per branch at each temperature counted as a replicate (*n* = 10). ResearchIR 4.0 software (FLIR, Wilsonville, OR, USA) was used to isolate the buds from the image and derive the surface temperature of the buds. These data were further analyzed in Matlab version R2018a (The MathWorks, Inc., 2018), where an emissivity map was created by compiling the single pixel emissivity of each bud (**Figure 2**). Emissivity map numerical data were used to generate the mean emissivity value for the CNC-coated and water control branch segments for each temperature. The cooling trends and treatment effect of emissivity values were compared using linear regression analysis in RStudio.

### 2.5 Scanning electron microscopy

#### 2.5.1 Dried films

**Figure 3:**
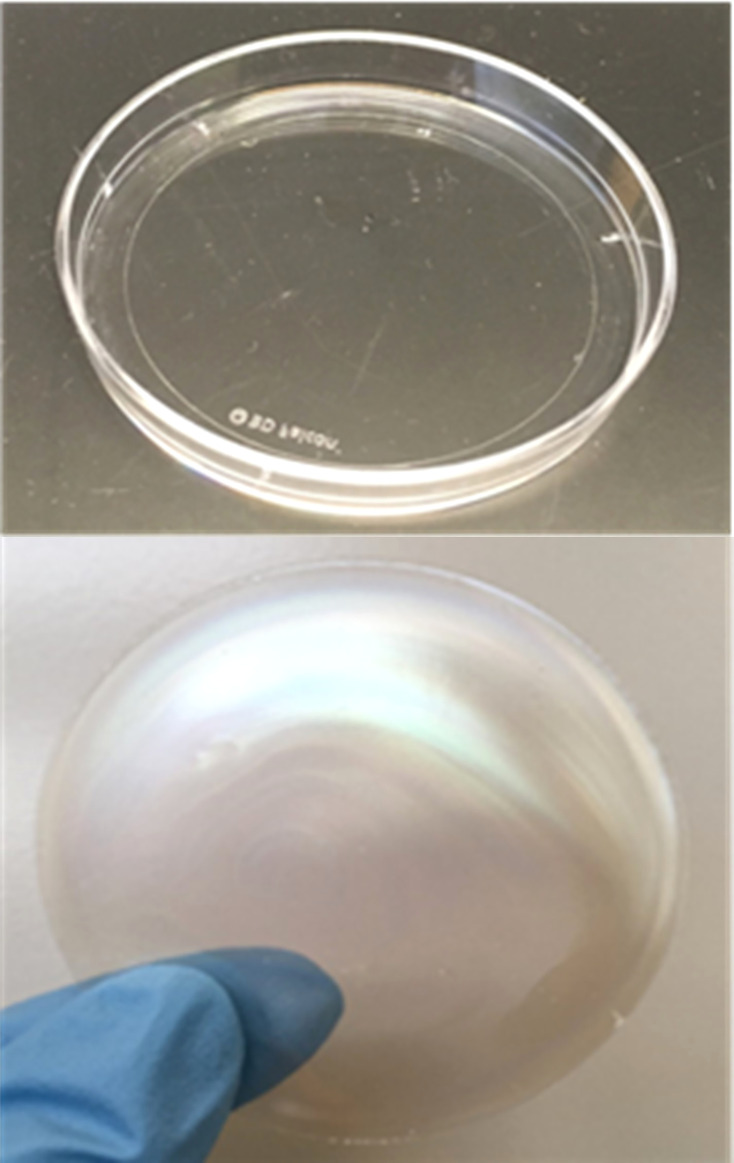
Example of wet cellulose nanocrystal dispersion evaporating in the Petri dish (top) and remaining cellulose nanocrystal film remaining after evaporation (bottom).

During a field application of 2% and 3% CNC dispersions, three Petri plates per treatment were hung in the tree canopy to collect the CNC dispersion. The water in the dispersion was allowed to evaporate in the lab for 24 h to form a solid film (**Figure 3**). The CNC film was measured with an SEM (Model Quanta 200F, FEI Company, Hillsboro, OR, USA) at an accelerating voltage of 20 kV at the Franceschi Microscopy and Imaging Center (FMIC), Washington State University, Pullman. Samples were examined at a magnification range of ×2,000 at 10 different positions of each film sample.

#### 2.5.2 Bud surface

Sweet cherry buds were treated with the 2% CNC dispersion 1, 2, and 3 days before imaging and stored at 0°C until imaging. Three buds per treatment and non-treated control buds were used for SEM analysis (Model Quanta 200F, FEI Company, Hillsboro, OR, USA) at the Franceschi Microscopy and Imaging Center (FMIC), Washington State University, Pullman. Buds were imaged at low (×35–×38) and high (×515–×623) magnifications.

## 3 Results

### 3.1 Calorimetry

**Figure 4:**
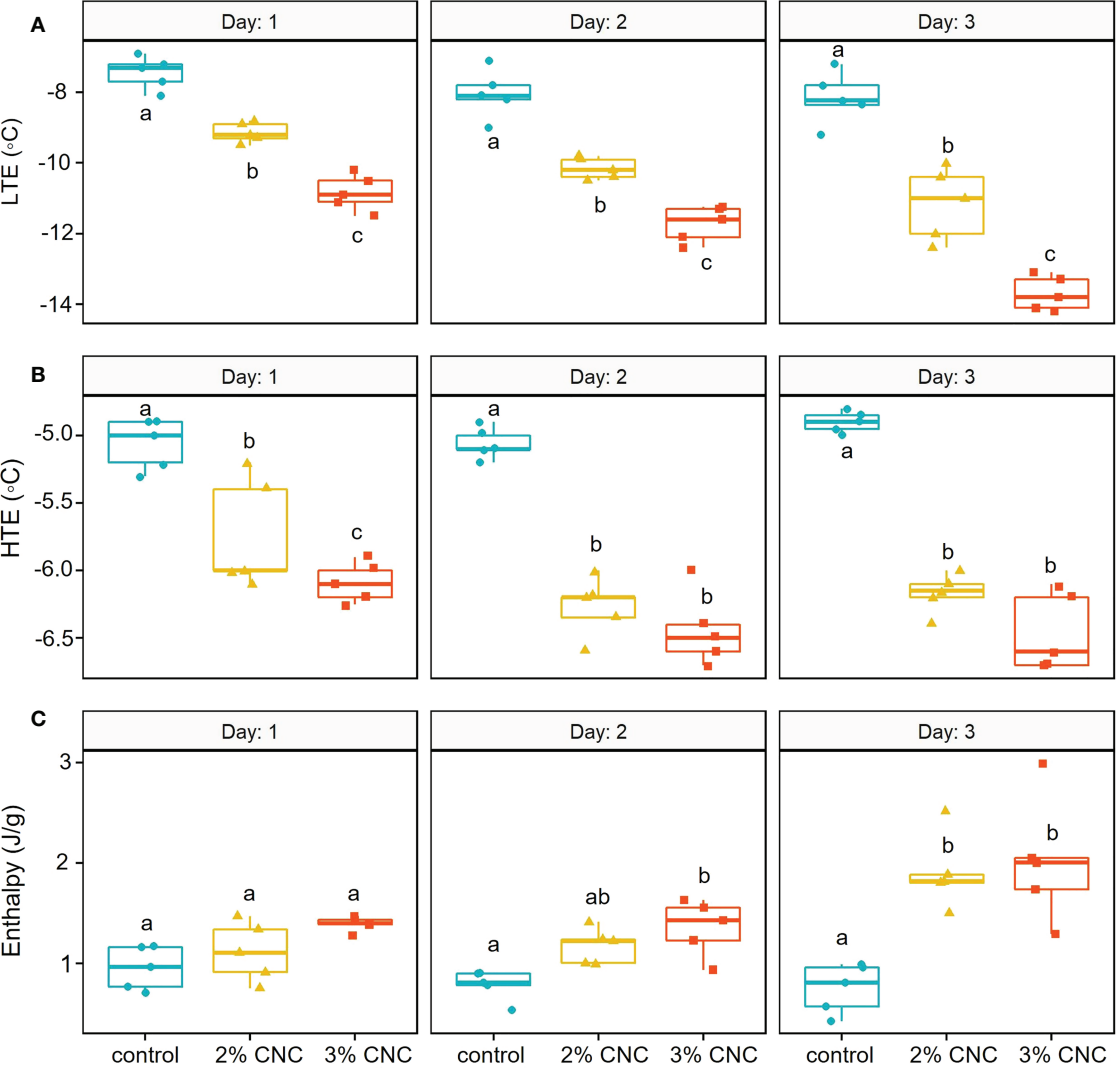
**(A)** Low-temperature exotherm (LTE) (°C), **(B)** high-temperature exotherm (HTE) (°C), and **(C)** enthalpy/latent heat capacity (J/g) of apple buds(n = 5) treated with 2% or 3% (wt) cellulose nanocrystal (CNC) dispersions or water control tested 1, 2, or 3 days post-application using digital scanning calorimetry (DSC). *Treatments with same letters do not differ significantly at α=0.05 with Tukey’s HSD Test.

At 1 day post-application, the mean LTE temperatures were −7.44°C ± 0.47°C, −9.14°C ± 0.29°C, and −10.84°C ± 0.51°C for the control and the 2% and 3% treated buds, respectively (**Figure 4A**). At this timepoint, we see that lethal freezing, as indicated by the LTE, was significantly delayed compared with the control by 1.7°C with 2% CNC coating (*p*< 0.0001) and 3.4°C with the 3% coating (*p*< 0.0001). Additionally, 3% CNC dispersions were able to delay internal freezing by 1.7°C more than the 2% (*p*< 0.0001). At 2 days post-application, mean LTE temperatures were −8.04°C ± 0.69°C, −10.16°C ± 0.30°C, and −11.73°C ± 0.51°C for the control and the 2% and 3% CNC, respectively (**Figure 4A**). At 2 days post-application, compared with the control, the CNC films were able to significantly delay LTE formation by 2.12°C (*p*< 0.0001) and 3.69°C (*p*< 0.0001) for the 2% and 3%, respectively. Here, the 3% CNC dispersion was able to delay internal freezing by 1.57°C more than the 2% (*p*< 0.0001). At 3 days post-application, the mean LTE temperatures were –8.16°C ± 0.74°C, −11.16°C ± 1.02°C, and −13.70°C ± 0.48°C for the control and the 2% and 3% treated buds, respectively (**Figure 4A**). At this timepoint, LTE was delayed by 3.00°C and 5.54°C for the 2% and 3% CNC dispersions, respectively, when compared with the control (*p*< 0.0001), with the 3% dispersion mean decreasing the LTE temperature by 2.54°C more than the 2% (*p*< 0.0001).

Non-lethal extracellular freezing, indicated by the HTE, was also delayed by the CNC dispersions at some time points (**Figure 4B**). At 1 day post-application, mean HTE temperatures were −5.06°C ± 0.18°C, −5.74°C ± 0.41°C, and −6.09°C ± 0.14°C for the water control and the 2% and 3% CNC-treated buds, respectively. HTE formation was slightly delayed in the CNC-treated buds when compared with the control, at a level of 0.68°C (*p*< 0.0001) and 1.03°C (*p*< 0.0001) for the 2% and 3%, respectively. The 0.35°C difference in HTE temperature between the 2% and 3% CNC treatment was also statistically significant (*p* = 0.040). For the buds tested 2 days post-application, the mean HTE temperatures were −5.05°C ± 0.11°C, −6.27°C ± 0.22°C, and −6.44°C ± 0.27°C for the water control and the 2% and 3% CNC-treated buds, respectively. When compared with the control, the treatments were able to decrease mean HTE by 1.22°C (*p*< 0.0001) and 1.39°C (*p*< 0.0001) for the 2% and 3%, respectively. However, on day 2, there was no significant difference in HTE between the two dispersion concentrations (*p* = 0.46). The mean HTE temperatures resulting from the DSC test performed 3 days post-application were −4.9°C ± 0.07°C, −6.17°C ± 0.15°C, and −6.46°C ± 0.29°C for the water control and the 2% and 3% CNC, respectively. Here, we see a significant delay in mean HTE temperature when compared with the control at 1.27°C (*p*< 0.0001) and 1.56°C (*p*< 0.0001) for the 2% and 3%, respectively.

In addition to HTE and LTE, the energy release curves also indicated the effect on latent heat capacity or enthalpy of the treatments (**Figure 4C**). On day 1, no statistical difference in enthalpy was seen between either treatment and the control (*p* = 0.6827; *p* = 0.0626) or between the two CNC concentrations (*p* = 0.3302). However, on day 2, mean enthalpy was 0.78 ± 0.15, 1.17 ± 0.18, and 1.35 ± 0.28 J/g for the water control, 2% dispersion, and 3% dispersion, respectively. Only the 3% dispersion treatment had statistically higher mean enthalpy when compared with the control, which increased by 0.57 J/g (*p* = 0.0099). Three days post-application, mean enthalpies were 0.75 ± 0.25, 1.90 ± 0.37, and 2.02 ± 0.62 J/g for the water control and the 2% and 3% CNC, respectively. Interestingly, 3 days post-application, both the 2% and 3% dispersions had significantly higher mean enthalpy values compared with the control. The 2% CNC had increased enthalpy by 1.15 J/g and the 3% CNC by 1.21 J/g.

### 3.2 Thermography

**Figure 5:**
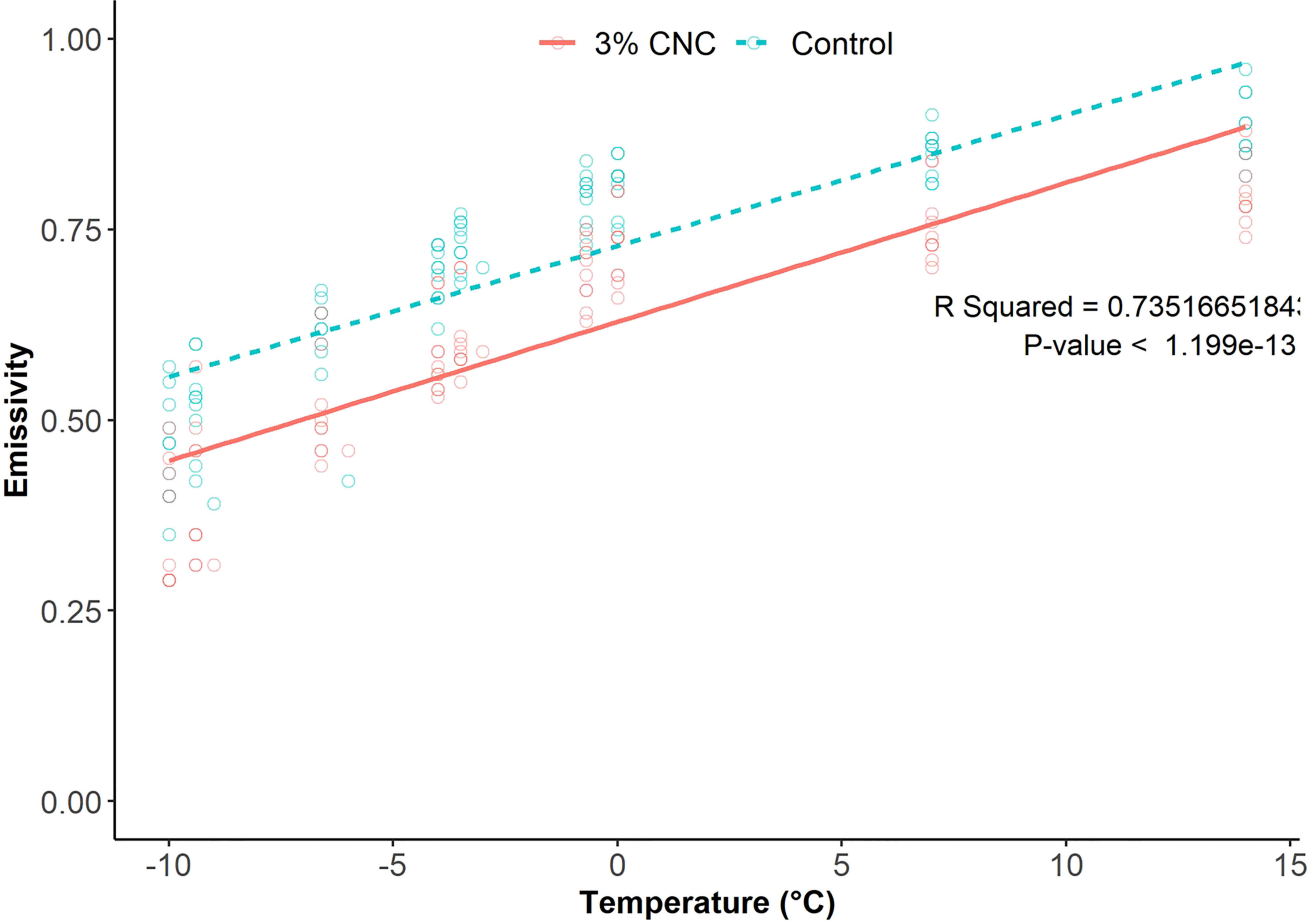
Plot and linear regression analysis of model-generated emissivity value from thermal images collected from cooling cherry fruit spurs (*n* = 10).

The emissivity of all buds decreased as air temperature decreased (**Figure 5**). The control buds had mean emissivity of 0.465 ± 0.07, 0.507 ± 0.07, 0.602 ± 0.07, 0.694 ± 0.04, 0.729 ± 0.03, 0.791 ± 0.03, 0.810 ± 0.03, 0.851 ± 0.03, and 0.884 ± 0.04 at temperatures of −10°C, −9.4°C, −6.6°C, −4°C, −3.5°C, −0.7°C, 0°C, 7°C, and 14°C, respectively. The buds treated with 3% CNC had mean emissivity of 0.353 ± 0.08, 0.396 ± 0.09, 0.506 ± 0.7, 0.584 ± 0.05, 0.608 ± 0.05, 0.694 ± 0.04, 0.728 ± 0.05, 0.755 ± 0.05, and 0.798 ± 0.04 at the same temperatures. The thermal camera images showed a mean decrease in emissivity of 16% in the CNC-coated buds compared with the control as temperature decreased (*p* = 1.199 · 10^−13^) (**Figure 5**).

### 3.3 Scanning electron microscopy

**Figure 6:**
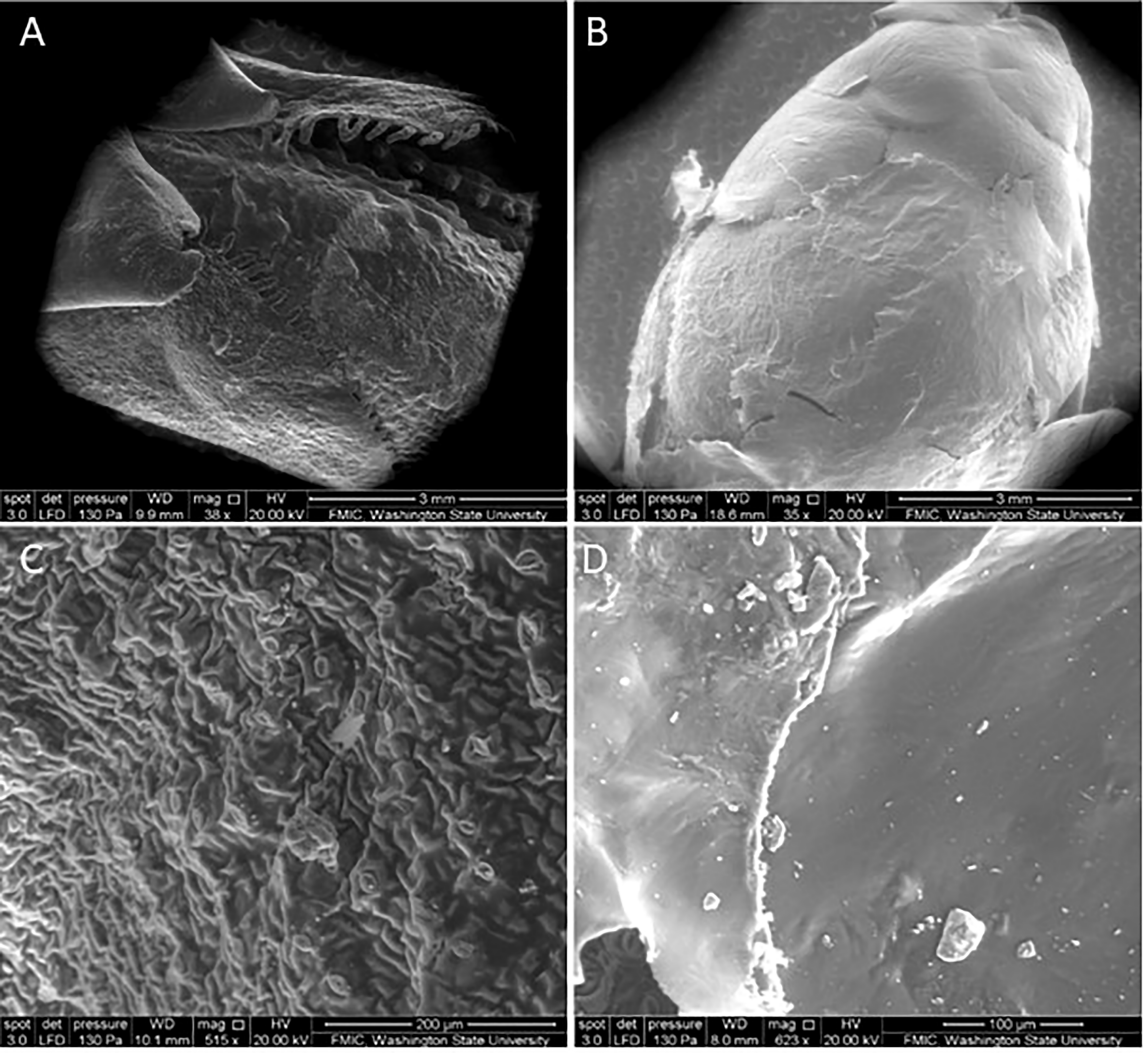
Scanning electron microscopy (SEM) micrographs of sweet cherry bud surface **(A)** non-treated at ×35 and **(B)** cellulose nanocrystal-treated at ×38 3 days post-application and **(C)** non-treated at ×515 and **(D)** cellulose nanocrystal-treated 3 days post-application at ×623.

**Figure 7:**
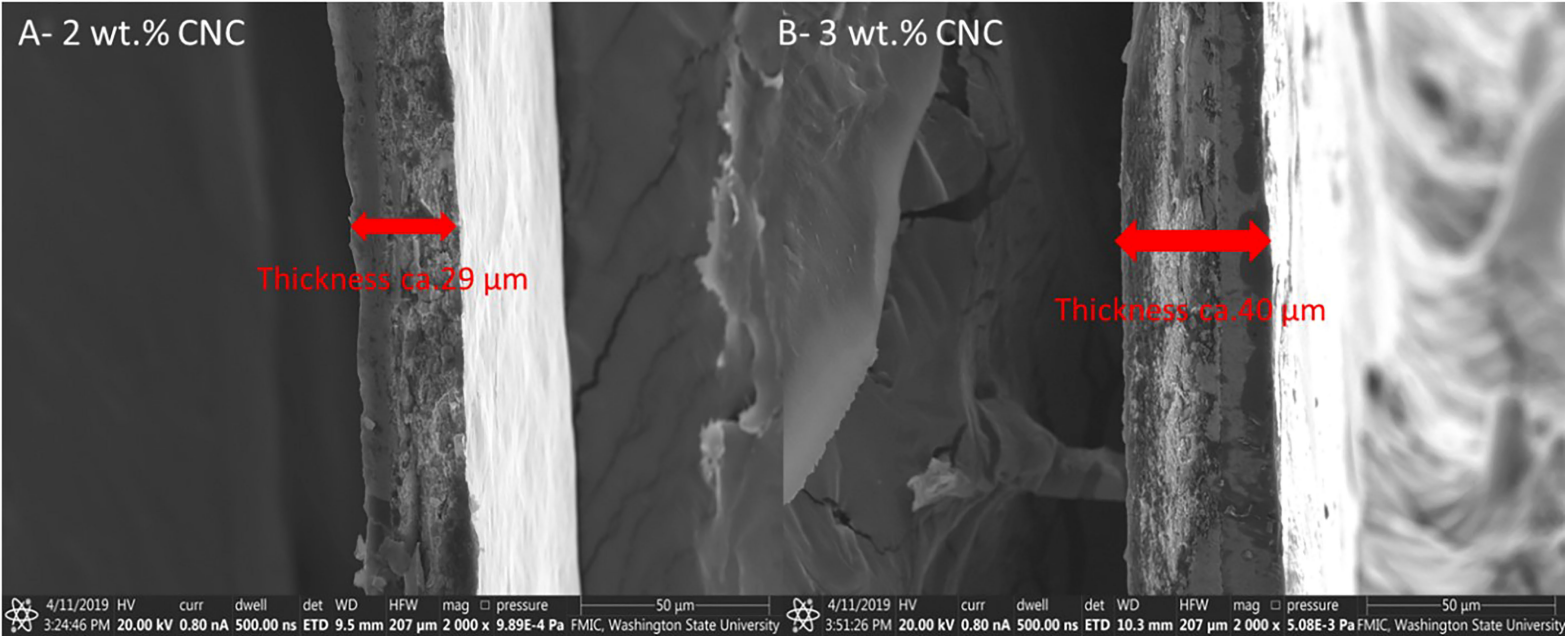
Scanning electron microscopy (SEM) micrographs of **(A)** 2% (wt) cellulose nanocrystal film (magnification ×2,000) and **(B)** 3% (wt) cellulose nanocrystal film (magnification ×2,000).

The SEM micrographs of the surface of the buds verified the presence of the nanofilms 3 days post-application (**Figure 6**). At low magnification, the CNC is visually noticeable by the change in texture seen on the bud surface (**Figure 6B**), compared with the non-treated (**Figure 6A**). At higher magnifications, the solid and rigid surface of the film becomes visible (**Figure 6D**), in contrast to the control (**Figure 6C**). The cross-sectional images of the film revealed the relationship between CNC concentration and film thickness. The films had a mean thickness of 29 and 40 µm for the 2% and 3%, respectively (**Figure 7**).

## 4 Discussion

Overall, all factors measured during the DSC tests were significantly affected by the CNC treatments, dispersion concentration, and time post-application. This was especially true in the lethal intracellular freezing, quantified by LTE temperatures. Compared with the control, buds treated with 2% CNC dispersions exhibited improved hardiness of mean LTE temperature by 1.7°C on day 1 and increased to 3.00°C on the third day. The 3% CNC dispersion showed a higher level of protection on day 1 (3.4°C), nearly double that of 2% CNC. Again, in the 3% dispersion, the ability to delay LTE formation increased over time. From day 1 to day 3, the mean LTE temperature of the control and buds treated with the 3% dispersion increased by 2.14°C to 5.54°C. These data demonstrate that lethal freezing within the CNC-treated buds was significantly lower (*p*< 0.0001) than in control buds. The decrease in LTE further demonstrates the previously described freeze protection qualities of CNC dispersions (Alhamid et al., 2018). It also suggests that the effect of a single application can be practical for as long as 3 days. This longevity is further supported by the verification of the films still present on the bud surface 3 days later with the SEM.

Similar to the LTE, HTE temperatures were decreased by the dispersion application and reduced over time. With HTEs, there was no significant difference between any treatments on day 1, but by day 3, the mean HTE was delayed by 1.3°C compared with the water control. The decrease in both the LTE and HTE suggests that ice formation is happening at lower temperatures. This decrease in ice nucleation temperature supports the insulative effect of CNC, meaning it is decreasing the rate of internal heat loss, causing internal water to freeze at a lower temperature. The CNC coating is keeping the internal temperatures of the treated buds higher during cooling likely accounting for the lower nucleation temperatures observed in the LTE and HTE of the CNC-coated buds. The higher concentration of the CNC (3%) provided up to a two-fold decrease in lethal temperature compared with the 2%. This high hardiness is likely due to the increased thickness of the films, improving their insulative effect. The enthalpy data further support the effect. Enthalpy was significantly higher in the treated buds, meaning more latent heat was released during nucleation. The difference in enthalpy in the coated buds suggests that less energy is being released into the environment during cooling. The enthalpy analyses also showed an increase in enthalpy over time in the treated buds, with the 3% treated buds increasing from 1.35 J/g on day 1 to 2.02 J/g by day 3. The increase in enthalpy over time, especially the magnitude, was surprising, as it was expected that a decline in protection can be observed if the coating was degrading.

The enthalpy of exotherms, as determined by DSC, has been shown to have a linear relationship with unfrozen water content (Vertucci, 1990). The increase in enthalpy suggests that more free water is present at the time of intracellular freezing. This implies that there could be a secondary mechanism involving water relations inside the buds. We offer two possible scenarios.

Firstly, DSC studies have shown that increases in enthalpy are correlated with the increasing unfreezable water (i.e., supercooled) in buds hardened to cold temperatures (Vertucci and Stushnoff, 1992; Sugiura et al., 1995), which verified the widely held belief that tolerance to cellular desiccation is integral to the ability to withstand freezing (Burke et al., 1976; Gusta et al., 2003). Therefore, the significant increase in enthalpy 3 days post-application could be due to the CNC films allowing the buds to supercool more water during the freezing process. Considering the nature of these coatings on the bud surface, as shown by the SEM, there could be inhibition of extrinsic nucleation caused by this coating. Materials like acrylic films (Glenn et al., 1998) and hydrophobic barriers (Wisniewski et al., 2002) have been shown to increase freezing tolerance in plant tissues by preventing ice nucleation from occurring on external nucleation sites like ice nucleation bacteria. This interference of external nucleation allows for more internal water to supercool, delaying the internal freezing of the tissue (Fuller et al., 2003). Based on the increase in supercooled water suggested by the increase in enthalpy in the DSC test, especially over time, and the decrease in nucleation temperature, this type of activity could also be taking place with these coatings. This is an area that deserves further investigation.

Alternatively, the effects of CNC on water regulation in plant tissues have been previously noted. A previous work showed decreased evapotranspiration to increase seed germination and longevity (Xu et al., 2020). A similar dispersion comprised of closely related materials, cellulose nanofibers, has even been implicated as an osmotic barrier to prevent cherry fruit cracking during a rain event dispersion (Jung et al., 2016). CNC films decreasing evapotranspiration would support the effects on enthalpy seen here if the decreased evapotranspiration is causing water to be retained in the buds.

The results of the DSC and SEM suggest that the CNC coatings are not degrading by day 3; in fact, the effect is increasing over time. This is similar to some field trials, in dry conditions, where the effect of the coating increases from 1 to 3 days post-application. However, in other trials, in wet conditions, we saw the coating degrade and lose efficacy rapidly (unpublished). This finding is not only indicative of the potential period of efficacy of these treatments but also reveals more about some of the biophysical and physiological effects of the CNC coatings.

The decrease in emissivity further supports the insulative effect of the CNC coatings on the reproductive buds of the tree fruit. Because the treated buds are radiating less thermal energy, internal cooling of the buds would occur at a proportionally decreased rate. This decrease in emissivity further adds evidence that an insulative effect is at least partially responsible for the decrease seen in both the LTE and HTE freezing temperatures. A previous work has also demonstrated the ability of other carbohydrate-based materials to lower the rate of heat loss in grape buds (Giuseppe et al., 2020). While a previous work has used thermography-derived emissivity to evaluate insulative materials (Wang et al., 2020) and the emissivity of plant leaves has been measured (Chen, 2015), this represents the first time thermography-derived emissivity has been used to evaluate insulative materials on plant tissues. The fact that lower emissivity was observed, which was also correlated with lower lethal freezing, establishes this technique as a method for evaluating plant-based dispersions or other frost-protectant materials. The results seen in the thermography study were also validated using a computational fluid dynamics modeling approach (Alhamid and Mo, 2021).

The 38% increase in thickness from the higher concentration seen in the SEM of the films may explain the trends seen in our DSC results, where concentration increased efficacy, especially over time. This would also be consistent with an insulative effect, where an increasing thickness increases the insulative value and possibly longevity.

This work was built on previous studies, which demonstrated a similar increase in lethal freezing temperature in dormant grape buds with DTA (Alhamid et al., 2018) by showing that this effect can last up to 3 days and even increase over time. This represents a significant advantage of CNC versus other previously described frost-protectant materials, which commonly degrade much more rapidly and often degrade entirely after just a single night (Choi et al., 1999). Having a material that can last at least 3 days from a single application would thus make the material more cost-effective. Very commonly, costs and labor associated with the application of a material limit the adoption of other materials (Snyder and Melo-Abreu, 2005).

Compared with other externally applied frost protection treatments (e.g., foams), CNC treatment may be advantageous. Unlike some foam materials that need a particular device to deploy, the CNC dispersions can be applied using application technologies already used by commercial growers. Additionally, the insulative foams are generally limited to lower-growing crops as they must fill the entire volume of the area needing protection. In contrast, CNC can be applied to the surface, making it a much more viable option in an orchard setting.

Our work here also suggests transferability across different species by demonstrating its efficacy in dormant apples and cherries and the previously shown dormant grape, which likely presented wide adaptability across temperate tree fruits and vines during dormancy (Alhamid et al., 2018). Additionally, these effects are likely transferable to other tissue types like floral tissue, shoots, and leaves. A previous work showed that CNC dispersions also effectively protected developing cherry flowers (Alhamid et al., 2018). CNC dispersion could likely be expanded even more widely to other crops like tender vegetables or citrus, where protecting foliar tissue is required.

The percent nanocrystal concentration of the dispersions significantly affected LTE and HTE internal freezing events in the apple buds tested under DSC. When combining the cross-sectional SEM images of the films, it was demonstrated that more concentrated dispersions created thicker films, showing that internal freezing generally is affected inside the buds, likely due to the decrease in heat loss as further evidenced by the reduction of emissivity seen in the thermography study of the treated and untreated cherry buds. This is an area worthy of further investigation—how a 38% increase in film thickness from 2% to 3% led to disproportionately greater improvements in cold tolerance. The effects of multiple layers (i.e., applications) of lower-concentration CNC dispersions *vs*. single applications of higher concentrations are similarly uninvestigated. The disproportional increases in effect, when compared to the increases in thickness, give further justification that coatings likely have dual actions aside from insulation as suggested above. Further large-scale field studies of CNC and other plant-based dispersions are required to better understand their utility at a commercial scale.

## 5 Conclusion

Here, we used several methods to further understand the effects and mechanisms of cellulose coatings on buds exposed to cold temperatures. We saw that lethal freezing temperature significantly lowered using DSC. The DSC also showed that the energy released during the exotherms increased with treatment and time, suggesting an additional mechanism involving water relations within the treated buds. This increase in water could be related to the ability of the coating to slow nucleation and increase the amount of supercooled water during freezing or from the coating’s ability to decrease evapotranspiration. SEM images were able to visually identify the CNC films on the epidermal tissues of the buds 3 days post-application. Additionally, the SEM images of the films confirmed that higher concentrated dispersions created thicker concentration films which likely explains the increase in effect with more concentrated dispersions. The thermography results showed that the emissivity of the treated buds was significantly decreased, further supporting the theory that CNC coatings have an insulative effect. Overall, these results further elucidate the mechanisms behind the protection of reproductive buds treated with cellulose nanocrystal dispersions. Additionally, the novel methodologies applied here can be used to further evaluate other plant-based dispersions or other materials for their frost protection ability. Further large-scale field studies of CNC and other plant-based dispersions are required to better understand their utility at a commercial scale.

## Data availability statement

The raw data supporting the conclusions of this article will be made available by the authors, without undue reservation.

## Author contributions

BA: conceptualization, methodology, data curation, statistical analysis, visualization, and writing—original draft. JA: conceptualization, methodology, software, data curation, and visualization. PW: material synthesis and SEM of buds. XZ: investigation, supervision, validation, and writing—review and editing. QZ: investigation, supervision, validation, and writing—review and editing. MW: investigation, supervision, validation, and writing—review and editing. All authors contributed to the article and approved the submitted version.

## Funding

This research was funded by the USDA National Institutes for Food and Agriculture (Accession No. 1015097) and the Washington State Tree Fruit Research Commission.

## Acknowledgments

The authors would like to acknowledge Dr. Lisa Neven and Dr. Rodney Cooper from the USDA Temperate Tree Fruit and Vegetable Research Unit, Wapato, WA, for allowing them to use and for their assistance with the calorimetry and thermography equipment.

## Conflict of interest

The authors declare that the research was conducted in the absence of any commercial or financial relationships that could be construed as a potential conflict of interest.

## Publisher’s note

All claims expressed in this article are solely those of the authors and do not necessarily represent those of their affiliated organizations, or those of the publisher, the editors and the reviewers. Any product that may be evaluated in this article, or claim that may be made by its manufacturer, is not guaranteed or endorsed by the publisher.
